# Network Pharmacology Interpretation of Fuzheng–Jiedu Decoction against Colorectal Cancer

**DOI:** 10.1155/2021/4652492

**Published:** 2021-02-20

**Authors:** Hongshuo Shi, Sisheng Tian, Hu Tian

**Affiliations:** ^1^College of Traditional Chinese Medicine, Shandong University of Traditional Chinese Medicine, Jinan, Shandong, China; ^2^School of Management, Shandong University of Traditional Chinese Medicine, Jinan, Shandong, China; ^3^College of Traditional Chinese Medicine, Shandong University of Traditional Chinese Medicine, Jinan, Shandong, China

## Abstract

**Introduction:**

Traditional Chinese medicine (TCM) believes that the pathogenic factors of colorectal cancer (CRC) are “deficiency, dampness, stasis, and toxin,” and Fuzheng–Jiedu Decoction (FJD) can resist these factors. In this study, we want to find out the potential targets and pathways of FJD in the treatment of CRC and also explain from a scientific point of view that FJD multidrug combination can resist “deficiency, dampness, stasis, and toxin.”

**Methods:**

We get the composition of FJD from the TCMSP database and get its potential target. We also get the potential target of colorectal cancer according to the OMIM Database, TTD Database, GeneCards Database, CTD Database, DrugBank Database, and DisGeNET Database. Subsequently, PPI analysis, KEGG pathways analysis, and GO biological processes analysis were carried out for the target of FJD in the therapy of colorectal cancer. In addition, we have also built a relevant network diagram.

**Results:**

In this study, we identified four core compounds of FJD in the therapy of colorectal cancer, including quercetin, kaempferol, beta-sitosterol, and stigmasterol. At the same time, we also obtained 30 core targets, including STAT3, INS, TP53, VEGFA, AKT1, TNF, IL6, JUN, EGF, CASP3, MAPK3, MAPK1, MAPK8, SRC, IGF1, CCND1, ESR1, EGFR, PTEN, MTOR, FOS, PTGS2, CXCL8, HRAS, CDH1, BCL2L1, FN1, MMP9, ERBB2, and JAK2. FJD treatment of colorectal cancer mainly involves 112 KEGG pathways, including FoxO (hsa04068) signaling pathway, PI3K-Akt (hsa04151) signaling pathway, HIF-1 (hsa04066) signaling pathway, T cell receptor (hsa04660) signaling pathway, and ErbB (hsa04012) signaling pathway. At the same time, 330 GO biological processes were summarized, including cell proliferation, cell apoptosis, angiogenesis, inflammation, and immune.

**Conclusions:**

In this study, we found that FJD can regulate cell proliferation, apoptosis, inflammation and immunity, and angiogenesis through PI3K-Akt signaling pathway to play an anti-CRC effect.

## 1. Introduction

Colorectal cancer (CRC), as the third most common cancer in the world, accounts for the fourth highest cancer mortality rate in the world [1]. It is a common malignant tumor of the digestive tract. Most patients' colorectal cancer is caused by their old age and unhealthy lifestyle, and only a few of them are caused by genetic factors. Other causes of colorectal cancer may include chronic inflammation of the gut, overweight, smoking, excessive intake of processed foods, and inadequate exercise [2]. In the diagnosis, due to the lack of effective clinical screening biomarkers, early symptoms of CRC are difficult to find. 15 to 25 percent of colorectal cancer patients have had liver metastasis at the time of detection, while 15 to 25 percent of CRC patients will be found with liver metastasis after radical surgery [3, 4]. At present, the main treatment methods for CRC include radiotherapy and chemotherapy, surgery, targeting, and immunotherapy [5, 6]. Because of its limitations, these treatments present the whole body toxicity, drug resistance, low selectivity, low efficacy, and other adverse reactions [7]. Therefore, we want to find a more effective and less toxic method against CRC [8].

Herbal medicine and traditional medical practice have been reported as a world upsurge by the World Health Organization's global Atlas of traditional, complementary, and alternative medicine [9]. Traditional Chinese medicine (TCM) has been used by the Chinese for thousands of years. It is a part of Oriental traditional medicine. It not only has the characteristics of good curative effect and small side effects but also has the overall and systematic philosophy [10]. Chinese herbal medicine treatment can control tumor growth, improve immune function, regulate tumor microenvironment, and play a significant role in preventing cancer relapse and transfer.

There are four main factors in TCM Syndromes of colorectal cancer: “deficiency, dampness, stasis, and toxin” [11]. According to the contemporary Chinese medicine masters, the treatment of tumors should be combined with tonifying deficiency and resisting evil [12]. In the early stage, we analyzed the literature and data mining of the experience of five batches of national famous experts in traditional Chinese medicine (500 in each batch, 3000 in total) and three sessions of Chinese Medicine Masters (30 students each, a total of 90 people) in the treatment of CRC. At the same time, we found the combination of the Fuzheng–Jiedu Decoction (FJD). FJD consists of *Panax ginseng* C.A. Mey (“RenShen” in Chinese, RS), *Pinellia ternata* (“BanXia” in Chinese, BX), and Smilacis Glabrae Rhizoma (“TuFuLing” in Chinese, TFL). Not only does the drug combination have a good antitumor effect, but also it can improve the symptoms of CRC such as asthenia and abdominal distension [13]. Based on the theory of TCM, the compatibility of FJD can not only treat “deficiency, dampness, stasis, and toxin” but also have mild efficacy and strong synergistic effect. RS can treat deficiency and improve immunity and antitumor, and BX and TFL can treat toxin, stasis, and dampness. Ginsenosides (TGCG) in RS guide generation cycle retardant and apoptosis in HT-29 cells [14]. BX can inhibit the hyperplasia of tumor cells, cut down the expression of tumor proteins, induce apoptosis of tumor cells, and act on multiple organ tumor cells [15]. Various compounds isolated from TFL have been proved to hold up the growth and hyperplasia of CRC cells in varying degrees [16]. Although the pharmacological research of RS, BX, TFL, and their monomers has made some achievements, the traditional pharmacological evaluation method is difficult to analyze the complex mechanism of FJD synergistic treatment of CRC. Therefore, this study through the network pharmacology elaborated its mechanism of action, hoping to provide the theoretical basis for the compatibility of TCM and provide more methods for the treatment of CRC.

**Figure 1:**
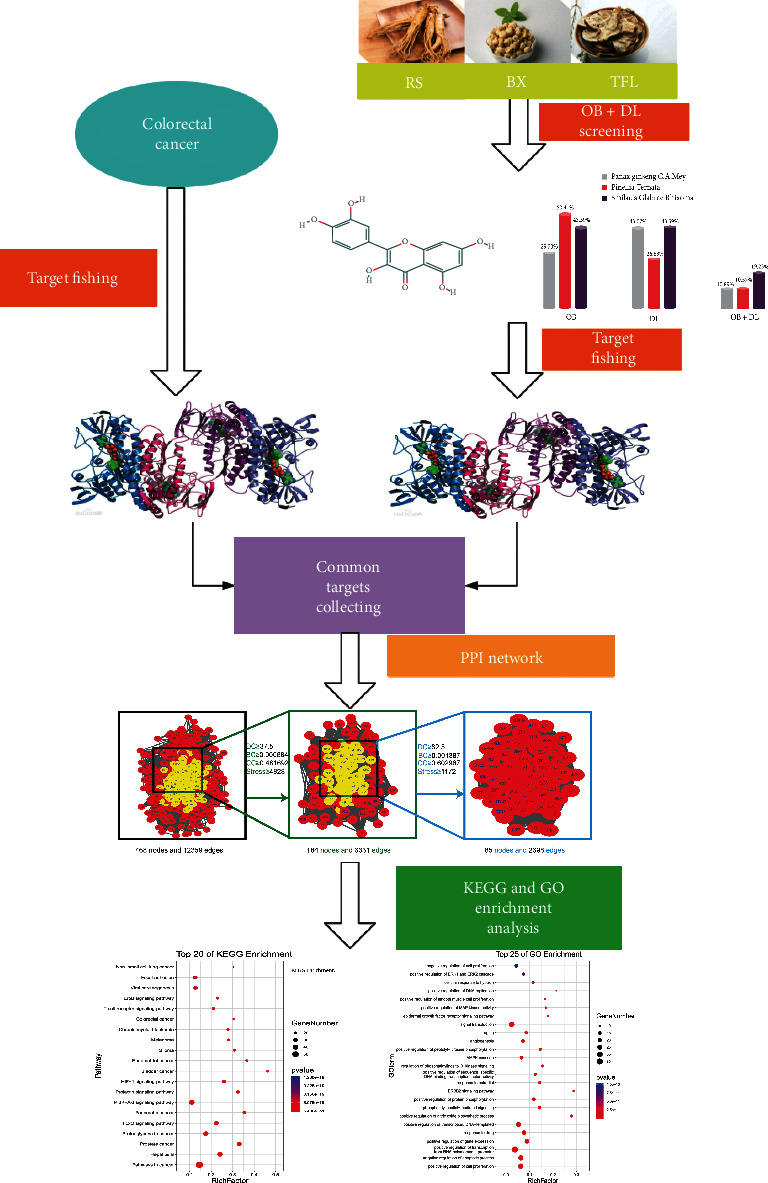
Flow chart of this network pharmacology research.

Network pharmacology is an effective new strategy integrating bioinformatics, chemical informatics, network biology, traditional pharmacology, and network analysis [17]. Network pharmacology, according to the model of “gene-target-disease,” systematically discusses the relationship between medicine and diseases, which accords with the holistic view of TCM theory [18]. In order to reveal the natural active compounds and potential mechanisms of traditional Chinese medicine prescriptions from a systematic and holistic perspective, network pharmacology provides a new method for them [19]. The goal of this study was to identify the natural compounds of FJD by network pharmacology and to explore the key target of FJD in the treatment of colorectal cancer, so as to comprehend its potential mechanism of action. Our plan is the expression in Figure 1.

## 2. Materials and Methods

### 2.1. Collection of Compounds in FJD

The compounds of RS, BX, and TFL in FJD were extracted from the TCM systems pharmacology (TCMSP) database. TCMSP database is one of the most comprehensive databases of Chinese herbal medicine, which is used to find the relationship among herbs, natural compounds, target protein, and diseases [20].

### 2.2. Screening of the Active Ingredients in FJD

Although FJD is composed of many natural compounds, only a few of them have pharmacological effects. Therefore, it is necessary to screen the pharmacokinetics of each compound of Chinese herbal medicine [21]. Based on the previously reported model, we screened various compounds in FJD according to the pharmacokinetic absorption (A), distribution (D), metabolism (M), and excretion (E) parameters. Based on the previous literature and the information in the relevant Chinese herbal medicine database, we selected natural compounds with oral bioavailability (OB) greater or equal to 30 percent and drug sensitivity (DL) greater or equal to 0.18 for further analysis [22] (Supplementary file 1).

### 2.3. Get the Target Protein of the Selected Compounds

All the active ingredients were input into the TCM systems pharmacology (TCMSP) database to get the known targets. The 2D structure of the compounds obtained from PubChem was imported into the SwissTargetPrediction database, and a threshold (probability >0.6) was set to obtain more credible targets for each compound.

### 2.4. Search Targets of CRC

We collected the CRC targets from six resources: (1) Online Mendelian Inheritance in Man database (OMIM); (2) Therapeutic Target Database (TTD); (3) GeneCards Human Gene database (score ≥ 30); (4) Comparative Toxicogenomics Database (CTD); (5) DrugBank Database; and (6) DisGeNET Database (Score_gda ≥0.3). The official names of the genes (FJD and CRC) were obtained as UniProt, and the species was selected as “Homo sapiens.” Then, the names of the targets were uniformly converted into gene and UniProt ID [23]. Finally, we map the target genes of FJD active constituent and CRC related target genes, and screen the coincident target genes as the related targets of FJD treatment for colorectal cancer.

### 2.5. PPI Network of Target Protein Interaction

The target gene of FJD for CRC was input into the STRING database, and its amplification and prediction were carried out. The network diagram of the target protein interaction in vivo was obtained. We first amplified the target protein of FJD against CRC with the STRING database and then input it into Cytoscape software. We use the “analysis network” tool in Cytoscape to get the protein interaction network to obtain the relevant parameters. Based on the four parameters of “Degree,” “BetweennessCentrality,” “ClosenessCentrality,” and “Stress,” we do topology analysis on the PPI network to get hub nodes [24].

### 2.6. Enrichment Analysis of GO and KEGG Pathway

GO biological process analysis and KEGG pathway analysis are effective methods to explain the role of target genes [25]. We input related hub target genes of FJD treatment CRC into DAVID 6.8 (high throughput functional labeling bioinformatics network platform) database for GO biological process analysis and KEGG pathway analysis and identified GO biological process with P less than or equal to 0.01 and KEGG pathway with P less than or equal to 0.01.

### 2.7. Construction of a Network of Herbs, Nature Compounds, Targets, and Pathways

On the basis of the compounds, targets, and pathways of FJD in the treatment of colorectal cancer, combined with the functions of Cytoscape 3.7.1 software, a network model of herbs, ingredients, targets, and diseases was constructed. According to this network model, we made a scientific analysis of the relationship between herbs, ingredients, targets, and diseases. We constructed (1) “compound-target” network diagram; (2) “herbal-compound-target” network diagram; (3) PPI network diagram of FJD potential targets for CRC; and (4) “pathway-target” network diagram. Nodes in the network diagram are linked by edges, and these nodes can represent herbs, compounds, targets, and KEGG pathways.

## 3. Results

### 3.1. Preparation by the Screening of the Natural Active Ingredients in FJD

**Figure 2:**
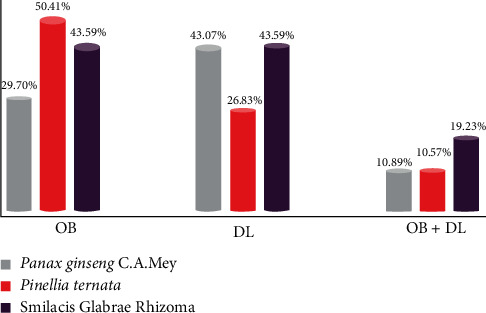
Column chart of composition screening.

According to the TCMSP database, there are 202, 123, and 78 compounds in FJD, respectively. These compounds were screened by OB greater than or equal to 30 percent and DL greater than or equal to 0.18. After filtering and deduplicating values, 46 potential compounds were included. There are 22, 13, and 15 compounds in RS, BX, and TFL, respectively. The composition screening diagram is shown in Figure 2. Among the 46 selected compounds, stigmasterol and beta-sitosterol were found in RS, BX, and TFL.

### 3.2. Study on the Target of Effective Compounds of FJD

**Figure 3:**
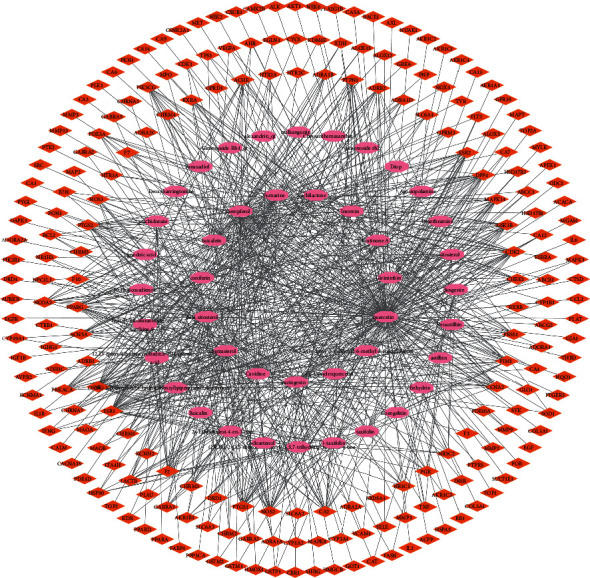
The “compound-target” network diagram of Fuzheng–Jiedu Decoction in the treatment.

**Figure 4:**
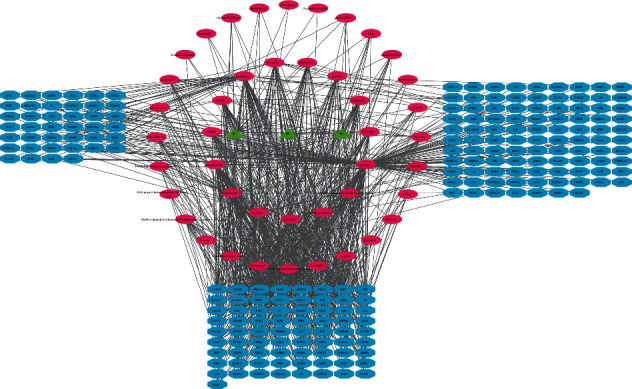
The “herbal-compound-target” network diagram of Fuzheng–Jiedu Decoction in the treatment of colorectal cancer. Green represents herbals. Pink represents the compounds. Blue represents the targets.

In this process, we screened 46 active compounds according to chemical similarity and got 212 related targets, including RS 128, BX 91, and TFL 188. As shown in Figure 3, we have established a “compound-target” network diagram composed of 252 nodes and 842 edges. We use the “analysis network” tool in Cytoscape to get the “Degree” parameter of the network, and we discover that the top four compounds of degree are quercetin (degree = 146), kaempferol (degree = 75), beta-sitosterol (degree = 51), and stigmasterol (degree = 45) (Table 1). As shown in Figure 4, we also built a “herbal-compound-target” network diagram composed of 256 nodes and 889 edges. Through this diagram, we can easily observe the relationship between herbs, ingredients, and targets and reveal the potential pharmacological effects of FJD. The conclusion of the network graph is consistent with the multitarget effect of Chinese herbal medicine and the synergistic effect of multidrug compatibility.

### 3.3. PPI Network Analysis

Through the method of multisource database integration, the CRC target data in OMIM, TTD, GeneCards, CTD, DrugBank, and DisGeNET databases are integrated. A total of 1018 CRC targets were obtained according to the screening and removal of duplicate values. The intersection of FJD and CRC related targets was collected and 58 common targets were obtained as the related targets of FJD in CRC.

**Figure 5:**
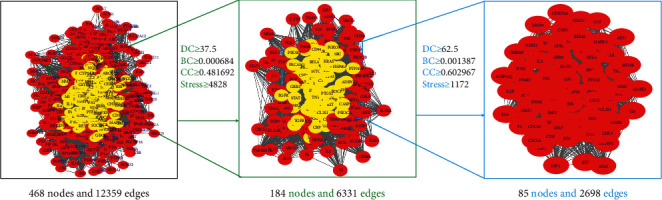
The process of topological screening for the PPI network.

**Figure 6:**
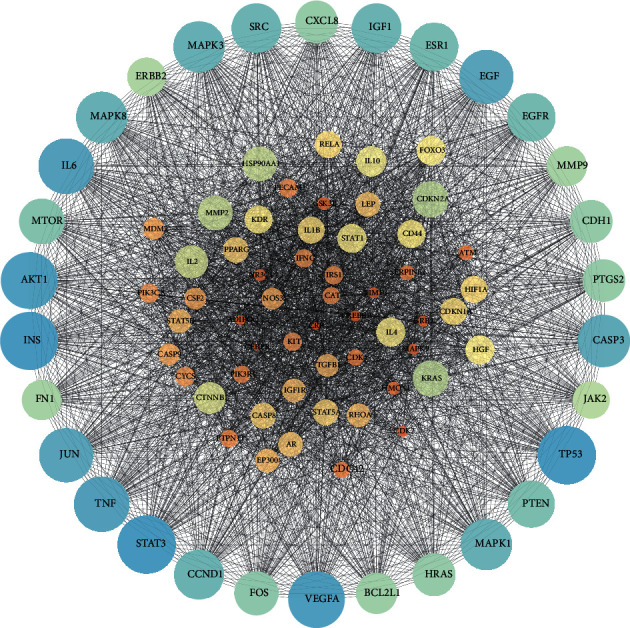
Construction of protein-protein interaction network expressed by hub target protein. 85 nodes represent 85 proteins, and 2698 edges represent the interaction between 2698 pairs of proteins. The node size and color represent the degree, and the data come from the string.

We input 58 target genes of FJD for colorectal cancer into the STRING database and got the PPI network diagram of 468 nodes. The obtained targets are divided into two parts, including 468 targets (58 public targets and 410 other human protein targets), which may represent FJD in in vivo response to CRC. There are nodes 468 and 12359 edges in total. Then, the topological characteristics of PPI are analyzed. We use the “analysis network” tool in Cytoscape to get the protein interaction network to obtain the relevant parameters, and based on the four parameters of “Degree,” “BetweennessCentrality,” “ClosenessCentrality,” and “Stress,” the index above the median value is selected as the key index [26] (Figure 5) and the core nodes of FJD acting on CRC are obtained. The threshold value of the first screening is “Degree” ≥37.5, “ClosenessCentrality” ≥0.481692, “BetweennessCentrality” ≥0.000684, and “Stress” ≥4828. Then, through the topological analysis data screening again, 85 hub targets are obtained. The criteria for screening were “Degree” ≥62.5, “ClosenessCentrality” ≥0.602967, “BetweennessCentrality” ≥0.001387, and “Stress” ≥1172. We got 85 hub nodes and established the related network diagram. The network diagram (Figure 6) was composed of 85 nodes and 2698 edges. By analyzing the diagram, the top 30 targets are STAT3, INS, TP53, VEGFA, AKT1, TNF, IL6, JUN, EGF, CASP3, MAPK3, MAPK1, MAPK8, SRC, IGF1, CCND1, ESR1, EGFR, PTEN, MTOR, FOS, PTGS2, CXCL8, HRAS, CDH1, BCL2L1, FN1, MMP9, ERBB2, and JAK2. These target genes (Table 2) may be the core targets of FJD in the treatment of CRC.

### 3.4. Enrichment Analysis of KEGG Pathway and GO Biological Process

After enrichment analysis of 85 hub targets by DAVID v6.8, we got 112 KEGG pathways and 330 GO biological processes according to *P* < 0.01.

#### 3.4.1. KEGG Pathway Analysis

**Figure 7:**
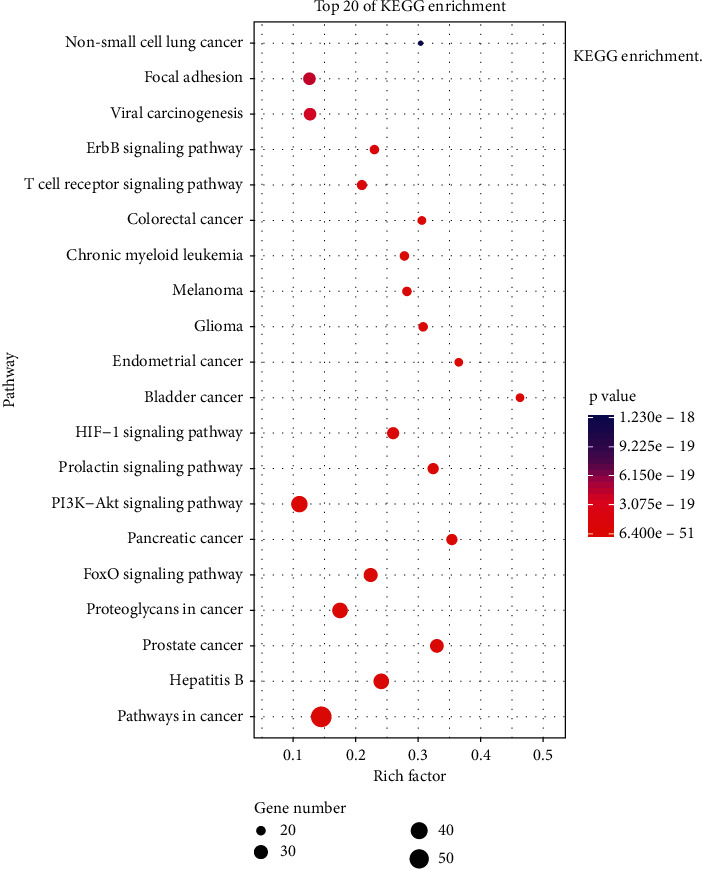
KEGG enrichment.

**Figure 8:**
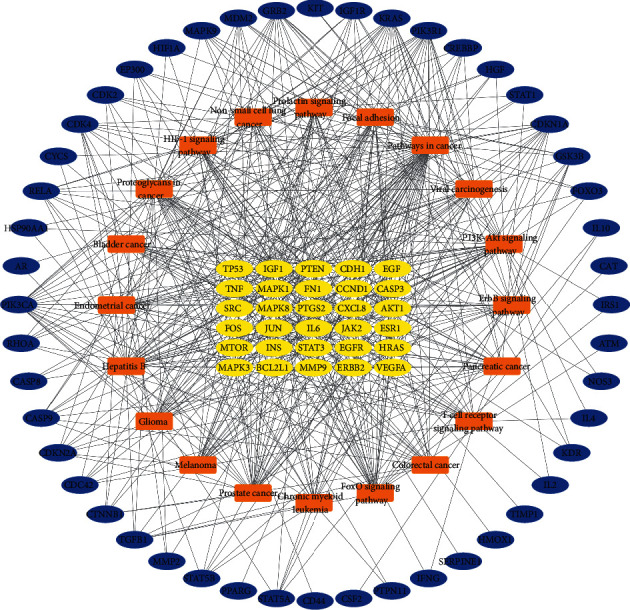
“Target-pathway” network. The yellow represents the core targets. The blue represents the other targets. The orange represents the related pathways.

According to the *P* value, we selected the first 20 KEGG (Figure 7) pathways for analysis. KEGG pathways, mainly FoxO (hsa04068) signaling pathway, PI3K-Akt (hsa04151) signaling pathway, HIF-1 (hsa04066) signaling pathway, T cell receptor (hsa04660) signaling pathway, and ErbB (hsa04012) signaling pathway. At the same time, we have constructed the network diagram between the first 20 KEGG pathways and 78 the targets. The network diagram (Figure 8) is composed of 98 nodes and 522 edges.

#### 3.4.2. Gene Ontology Analysis

**Figure 9:**
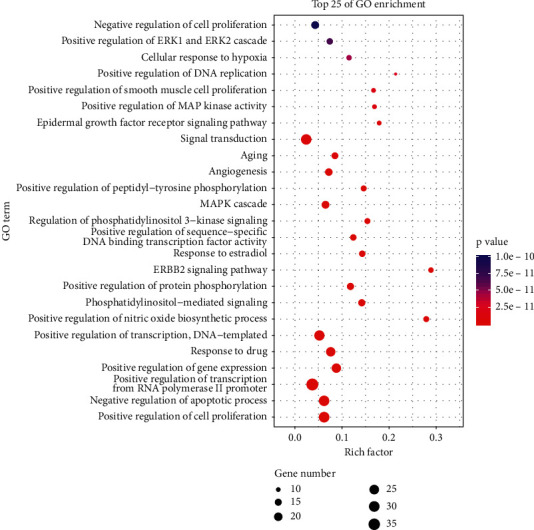
GO biological processes enrichment.

According to the *P* value, we selected the first 25 GO biological processes (Figure 9) for analysis, and the top 10 are (GO: 0008284) positive regulation of cell proliferation, (GO: 0043066) negative regulation of apoptotic process, (GO: 0045944) positive regulation of transcription from RNA polymerase II promoter, (GO: 0010628) positive regulation of gene expression, (GO: 0042493) response to drug, (GO: 0045893) positive regulation of transcription, (GO: 0045429) positive regulation of nitric oxide biosynthetic process, (GO: 0048015) phosphatidylinositol-mediated signaling, (GO: 0001934) positive regulation of protein phosphorylation, and (GO: 0038128) ERBB2 signaling pathway.

## 4. Discussion

As the third most common cancer, colorectal cancer patients are less likely to survive for more than five years, still less than 10% [25, 27]. Chinese herbal medicine has achieved an ideal curative effect in the treatment of tumors. However, it is difficult to explain the complex mechanism of the traditional method. Therefore, the integration of network pharmacology based on big data bioinformatics into the molecular mechanism of Chinese herbal medicine in the treatment of diseases is of great significance.

According to the network diagram of “compound-target,” we got 4 core compounds, namely, quercetin, kaempferol, baicalein, and beta-sitosterol. Quercetin is a kind of polyglycol hydroxyflavonoid widely existing in nature. It has many functions such as antitumor, antioxidation, anti-inflammatory, and immune regulation [28]. In vitro experiments have shown that quercetin used alone or in combination with other drugs can inhibit the development of CRC tumors by blocking the division cycle of cancer cells, reducing the biological activity of cancer cells, and regulating certain cancer-related signaling pathways [29]. Kaempferol exists in propolis, propolis tea, and vegetables. It is a common flavonoid in food. It has the effect of antiproliferation and inducing apoptosis of human colon cancer cells [30]. Beta-sitosterol is one of the most abundant phytosterols in the diet, comparable to animal cholesterol [31]. It has been reported that *ß*-sitosterol can significantly inhibit the progression of DMH-induced CRC in mice [32]. Modern research shows that stigmasterol can destroy the formation of tumor blood vessels in vivo [33]. These compounds are the material basis of FJD acting on CRC.

According to the analysis of KEGG enrichment, FJD treatment of CRC may be achieved through multiple pathways, the most important of which may be PI3K/Akt single pathway. After enrichment analysis of KEGG pathways and GO biological process, we found that the potential mechanism of FJD in the treatment of CRC may be related to its participation in cell proliferation, cell apoptosis, inflammation and immune, and angiogenesis. Next, we will elaborate on these four aspects in combination with the core targets.

### 4.1. Cell Proliferation

PI3K/Akt signaling pathway is critical for the proliferation of colorectal cancer cells. Among them, PI3K/Akt is an important intracellular signaling pathway, which is closely related to the formation and progress of colorectal cancer. It can participate in cell growth, proliferation, differentiation, and migration. PI3K gene activation and PTEN inactivation common in CRC lead to Akt and overexpression of downstream targets, including PKC, promotes cell growth and rescues cells from apoptosis. Therefore, the inhibition of PI3K/Akt has been widely used to treat CRC [34, 35]. EGF binds with EGFR, thus activating the epidermal growth factor receptor, activating PI3K/Akt pathway, and finally promoting tumor proliferation [36, 37]. Jun transcription factor (JUN) is a key gene of the AP-1 family of transcription factor complexes. MiR-22 is considered to be an important tumor suppressor miRNAs [38, 39], which can significantly inhibit the ability of AP-1 to bind DNA [40]. According to reports, Jun can downregulate the expression of p53 (TP53), while p53 can activate the expression of miR-22 [41]. Some results show that the overexpression of proto-oncogenes in fibroblasts makes it easy for c-jun (JUN) to bind to cyclin D1 (CCND1), thereby activating the expression of cyclin D1 and promoting the cell cycle process [42]. Cell division cycle-associated protein 2 may target CCND1 by promoting PI3K/Akt pathway, thus promoting the abnormal proliferation of colorectal cancer cells [43]. IGF1 (insulin-like growth factor I) is a powerful mitogenic factor in CRC cells and an important antiapoptotic factor in CRC cells. IGF1 can bind to its related receptor IGF1R to induce PI3K/Akt pathway. As we all know, the liver is the most common metastatic site of CRC. The source of IGFI is the liver. Therefore, inhibiting IGF1R may be an effective method for the treatment of colorectal cancer [44].

Tumor cell proliferation plays a key role in the occurrence and development of human malignant tumors. Quercetin is derived from Chinese herbal medicine TFL, which can act on four targets: JUN, MAPK1, EGF, and EGFR. It may be the main compound of FJD to inhibit cancer cell proliferation. It has been reported that quercetin can inhibit proliferation and induce apoptosis of SW480, HCT116, HT 29, Caco-2, LoVo, and other CRC cells [45]. Also, baicalein (Table 1) can inhibit MAPK, Akt, or mTOR to hold up the abnormal proliferation of cancer cells.

### 4.2. Cell Apoptosis

Tumor suppressor p53 (TP53) can control the progression of cancer by inducing apoptosis, upregulating DNA repair protein, and maintaining genome stability [46, 47]. At the same time, p53 mutants can activate the Akt signal to promote cancer cell invasion. Integrin and EGFR depend on the circulation of RCP to participate in this process [48]. In addition, in many cancers, including colorectal cancer, the expression of cyclooxygenase-2 (PTGS2) is out of control, while the expression of cyclooxygenase-2 is negatively regulated by p53 [49]. As an inhibitor, p53 inhibits the expression of some antiapoptotic genes, including Bcl-2 and promotes apoptosis [50]. Bcl-2 is an apoptotic inhibitor protein, and BCL2L1 belongs to Bcl-2 protein family [51]. Bax is an apoptotic protein, which can induce the formation of apoptotic bodies and upregulate the expression of Caspase-3 (CASP3). BCL2L1 can steady the chondriosome localization of Bax and make it lose its biological activity [52]. These proteins are the regulators of apoptosis in PI3K-Akt pathway [53]. It has been shown that CXCL8 has a negative correlation with the expression of Bcl-2-related cell death protein (BAD), thus inhibiting the apoptosis of colorectal cancer cells [54]. Besides, mir-204 may inhibit the expression of CXCL8 by inhibiting PI3K/Akt/mTOR pathway [55]. Also, the most important way FOXO participates in apoptosis is PI3K/Akt pathway, which triggers the expression of death receptor ligands such as TNF apoptosis ligand and Bcl-2 family members [56].

The disorder of apoptosis is directly or indirectly related to the occurrence of tumors [57]. In FJD, the compounds acting on TP53 are quercetin, baicalein, and diosgenin. The results show that baicalein can inhibit angiogenesis [58] and prevent tumor metastasis [59], and it can control the abnormal proliferation and cell cycle of cancer cells by inhibiting Ezrin and activating p53 pathway-related proteins [60]. Besides, a combination of kaempferol and 5-fluorouracil induces apoptosis through PI3K/Akt signaling pathway [61]. Studies show that quercetin-induced apoptosis inhibits PI3K/Akt and apoptosis inhibitor Bcl-2, and the accumulation of HIF-1*α* also has an inhibitory effect [62]. Diosgenin (Table 1) can upregulate p38 MAPK pathway-related proteins, thus promoting TRAIL (a member of the TNF cytokine family) induced apoptosis [63].

### 4.3. Inflammation and Immune

Patients with inflammatory bowel disease (IBD) including ulcerative colitis have a higher probability of developing colitis-related CRC [64]. During inflammation, immune cells can lead to the production of highly genotoxic oxygen/nitrogen-reactive substances, such as IL-6 and TNF-*α* [6], and form an inflammatory microenvironment. The production of these inflammatory factors is mediated through the following signaling pathways, such as signal transducers and transcription activator 3 (STAT3), PI3K/AKT, cyclooxygenase-2 (PTGS2), and JAK/STAT. IL-6 binds to heterodimer receptors, thereby activating gp130 on the surface of cell membranes [65], activating and maintaining the phosphorylation level of STAT3, and promoting cell proliferation, migration, invasion, and antiapoptosis [66]. At the same time, TNF-*α* plays a biological role in tumor cells through the STAT3 signaling pathway, so the STAT3 signaling pathway is a bridge between inflammation and tumor [67]. PTGS2 encodes inducible prostaglandin synthase cyclooxygenase-2, which is an important rate-limiting enzyme produced by prostaglandins and has a key significance for malignant tumors. Activation of the PI3K/Akt signaling pathway can upregulate PTGS2 [68], which has been proved to be involved in the production of inflammatory prostaglandins through stimulation events and in various biological processes such as tumor cell proliferation, angiogenesis, and invasion [69]. T cell mediated acquired immunity can effectively promote the process of tumor regression. Tumor cells affect immune cells through the STAT3 signaling pathway and participate in the immune escape of tumor cells. Therefore, STAT3 has become an important protein in immunotherapy research [70]. IL-6 produced by the tumor-bearing host can aggravate the immunosuppression in the tumor microenvironment by inhibiting antitumor cells including CD8 + T cells, thus leading to the metastasis and colonization of colon cancer cells [71].

Chronic inflammation is an important factor leading to CRC. TNF gene is regulated by quercetin, kaempferol, astilbin, and ginsenoside rh2 in FJD and IL6 gene is regulated by quercetin. Quercetin has a strong anti-inflammatory effect. It can reduce some special inflammatory factors including TNF-*α*, by preventing the phosphorylation and degradation of I*κ*B in the process of inflammation [72]. Studies have shown that ginsenoside rh2 (Table 1) upregulates the secretion levels of TNF-*α* and IL-6 in the serum of colon cancer mice, relieves tumor-related immunosuppression, and prolongs the survival of colon cancer mice [73].

### 4.4. Angiogenesis

VEGFA can induce angiogenesis by promoting the abnormal proliferation and migration of vascular endothelial cells, which is conducive to the growth, invasion, and metastasis of tumor [74]. Hypoxia is the feature of the tumor microenvironment. Cells under hypoxia can produce hypoxia inducible factor-1 *α* (HIF-1 *α*), which can lead to the enhancement of tumor cell invasion and metastasis [35]. Under hypoxia state, VEGF enhanced expression and even the initial starting factor of angiogenesis in tumor tissue, while HIF-1*α* played a “bridge” role in the process from hypoxia to VEGF increased expression [75]. Under the condition of hypoxia, it has also been found that HIF-1*α* can also combine with the hypoxia response element (HRE) of hypoxia response gene, promote the transcription of the downstream target gene to enter the nucleus, promote Akt activation, activate PI3K/Akt signal transduction pathway, inhibit endothelial cell apoptosis, and promote angiogenesis [76].

As we all know, angiogenesis is a necessary condition and a key step for tumor growth and metastasis. Therefore, the inhibition of tumor angiogenesis can block the occurrence, development, invasion, and metastasis of tumors to a certain extent [77]. In FJD, the main compounds of genes that act on VEGFA are baicalein, diosgenin, and quercetin. Quercetin can inhibit tumor angiogenesis by inhibiting VEGFR2. Also, some studies have shown that kaempferol can inhibit the HIF-1*α* and VEGFR2 in endothelial cells through PI3K/Akt/mTOR signals, thus showing its antiangiogenesis ability [78].

## 5. Conclusion

Traditional Chinese medicine has been passed down for thousands of years in China. The idea of Chinese herbal medicine treating tumors is the combination of tonifying deficiency and resisting evil. Based on the combination of modern medicine, a lot of experience in treating malignant tumors has been summarized. In this study, we first recognized from the perspective of network pharmacology that FJD regulates cell proliferation, apoptosis, inflammation, immunity, and angiogenesis through the PI3K-Akt signaling pathway. In addition, we also provide a scientific explanation for FJD in treating “deficiency, dampness, stasis, and toxin,” and we hope to open a window for understanding the complex mechanism of action of Chinese herbal medicine and the essence of more concepts of traditional Chinese medicine.

## Figures and Tables

**Table 1:** Compound information sheet.

Molecule ID	Molecule name	Structure	OB (%)	DL	Herb
Quercetin	MOL000098	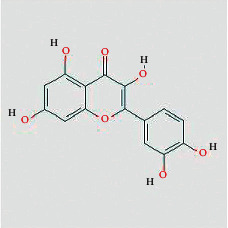	46.43	0.28	TFL
Kaempferol	MOL000422	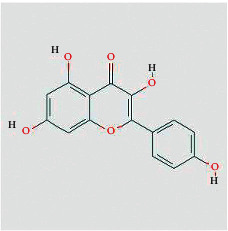	41.88	0.24	RS
Beta-sitosterol	MOL000358	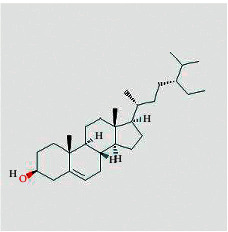	36.91	0.75	RS, BX, and TFL
Stigmasterol	MOL000449	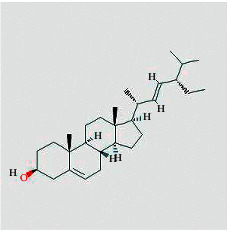	43.83	0.76	RS, BX, and TFL
Diosgenin	MOL000546	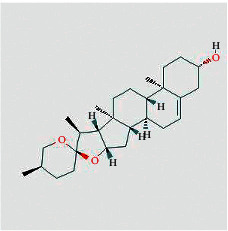	80.88	0.81	TFL
Ginsenoside rh2	MOL005344	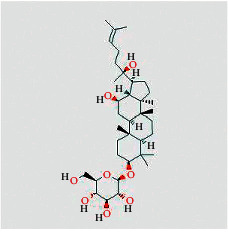	36.32	0.56	RS
Baicalein	MOL002714	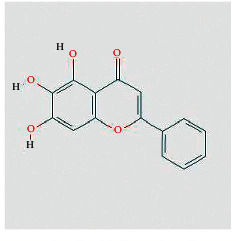	33.52	0.21	BX

**Table 2:** Information on 30 core targets.

Gene	Description	UniProt	Degree	Source
STAT3	Signal transducer and activator of transcription 3	P40763	84	Predicted
INS	Insulin	P01308	84	Predicted
TP53	Cellular tumor antigen p53	P04637	84	Validated
VEGFA	Vascular endothelial growth factor A	P15692	83	Validated
AKT1	RAC-alpha serine/threonine-protein kinase	P31749	83	Predicted
TNF	Tumor necrosis factor	P01375	82	Validated
IL6	Interleukin-6	P05231	82	Validated
JUN	Transcription factor AP-1	P05412	81	Validated
EGF	Pro-epidermal growth factor	P01133	81	Validated
CASP3	Caspase-3	P42574	80	Predicted
MAPK3	Mitogen-activated protein kinase 3	P27361	79	Predicted
MAPK1	Mitogen-activated protein kinase 1	P28482	79	Validated
MAPK8	Mitogen-activated protein kinase 8	P45983	79	Validated
SRC	proto-oncogene tyrosine-protein kinase Src	P12931	78	Predicted
IGF1	Insulin-like growth factor I	P05019	78	Predicted
CCND1	G1/S-specific cyclin D1	P24385	78	Predicted
ESR1	Estrogen receptor	P03372	77	Validated
EGFR	Epidermal growth factor receptor	P00533	77	Validated
PTEN	Phosphatase and tensin homolog	P60484	76	Predicted
MTOR	Serine/threonine-protein kinase mTOR	P42345	75	Predicted
FOS	Proto-oncogene c-Fos	P01100	74	Predicted
PTGS2	Prostaglandin G/H synthase 2	P35354	73	Validated
CXCL8	Interleukin-8	P10145	73	Predicted
HRAS	GTPase HRas	P01112	73	Predicted
CDH1	Cadherin-1	P12830	73	Predicted
BCL2L1	Bcl-2-like protein 1	Q07817	72	Predicted
FN1	Fibronectin	P02751	71	Predicted
MMP9	Matrix metalloproteinase-9	P14780	71	Predicted
ERBB2	Receptor tyrosine-protein kinase erbB-2	P04626	70	Predicted
JAK2	Tyrosine-protein kinase JAK2	O60674	69	Predicted

## Data Availability

The data used to support the findings of this study are available from the corresponding author upon request. The authors got the composition of FJD from the TCMSP database (http://tcmspw.com/tcmspsearch.php) and its potential target from the TCMSP database (http://tcmspw.com/tcmspsearch.php) and Swiss TargetPrediction database (http://www.swisstargetprediction.ch/). They also got the potential target of colorectal cancer according to the OMIM Database (http://www.omim.org/), TTD Database (http://bidd.nus.edu.sg/group/cjttd/), GeneCards Database, CTD Database (http://ctdbase.org/), DrugBank Database (https://www.drugbank.ca/), and DisGeNET Database (http://www.disgenet.org/home/). Subsequently, PPI analysis (STRING Database, https://string-db.org/), KEGG pathways (DAVID Database, https://david.ncifcrf.gov/) analysis, and GO biological processes (DAVID Database, https://david.ncifcrf.gov/) analysis were carried out for the target of FJD in the therapy of colorectal cancer.

## Supplementary Materials

Supplementary Materials Supplementary File 1 includes natural compounds contained in the Fuzheng–Jiedu Decoction (FJD), and the ADME parameter is oral bioavailability (OB) ≥ 30% and drug-likeness (DL) ≥ 0.18. Table (A) in Supplementary File 1 indicates the compounds in RS; Table (B) in Supplementary File 1 indicates the compounds in BX; and Table (C) in Supplementary File 1 indicates the compounds in TFL.
