# Supplementary material for: Network Pharmacology Interpretation of Fuzheng–Jiedu Decoction against Colorectal Cancer
Source: Evid Based Complement Alternat Med. 2021 Feb 20;2021:4652492. doi: 10.1155/2021/4652492 (PMC7914091; doi:10.1155/2021/4652492)
Supplement: Supplementary Materials — Supplementary File 1 includes natural compounds contained in the Fuzheng–Jiedu Decoction (FJD), and the ADME parameter is oral bioavailability (OB) ≥ 30% and drug-likeness (DL) ≥ 0.18. Table (A) in Supplementary File 1 indicates the compounds in RS; Table (B) in Supplementary File 1 indicates the compounds in BX; and Table (C) in Supplementary File 1 indicates the compounds in TFL. [file 4652492.f1.docx]

| RS-Mol ID | Molecule Name | OB (%) | DL |
| --- | --- | --- | --- |
| MOL002879 | Diop | 43.59 | 0.39 |
| MOL000449 | Stigmasterol | 43.83 | 0.76 |
| MOL000358 | beta-sitosterol | 36.91 | 0.75 |
| MOL003648 | Inermin | 65.83 | 0.54 |
| MOL000422 | kaempferol | 41.88 | 0.24 |
| MOL004492 | Chrysanthemaxanthin | 38.72 | 0.58 |
| MOL005308 | Aposiopolamine | 66.65 | 0.22 |
| MOL005314 | Celabenzine | 101.88 | 0.49 |
| MOL005317 | Deoxyharringtonine | 39.27 | 0.81 |
| MOL005318 | Dianthramine | 40.45 | 0.2 |
| MOL005320 | arachidonate | 45.57 | 0.2 |
| MOL005321 | Frutinone A | 65.9 | 0.34 |
| MOL005344 | ginsenoside rh2 | 36.32 | 0.56 |
| MOL005348 | Ginsenoside-Rh4_qt | 31.11 | 0.78 |
| MOL005356 | Girinimbin | 61.22 | 0.31 |
| MOL005357 | Gomisin B | 31.99 | 0.83 |
| MOL005360 | malkangunin | 57.71 | 0.63 |
| MOL005376 | Panaxadiol | 33.09 | 0.79 |
| MOL005384 | suchilactone | 57.52 | 0.56 |
| MOL005399 | alexandrin_qt | 36.91 | 0.75 |
| MOL005401 | ginsenoside Rg5_qt | 39.56 | 0.79 |
| MOL000787 | Fumarine | 59.26 | 0.83 |

(A): Compounds in RS

| BX-Mol ID | Molecule Name | OB (%) | DL |
| --- | --- | --- | --- |
| MOL001755 | 24-Ethylcholest-4-en-3-one | 36.08 | 0.76 |
| MOL002670 | Cavidine | 35.64 | 0.81 |
| MOL002714 | baicalein | 33.52 | 0.21 |
| MOL002776 | Baicalin | 40.12 | 0.75 |
| MOL000358 | beta-sitosterol | 36.91 | 0.75 |
| MOL000449 | Stigmasterol | 43.83 | 0.76 |
| MOL005030 | gondoic acid | 30.7 | 0.2 |
| MOL000519 | coniferin | 31.11 | 0.32 |
| MOL006936 | 10,13-eicosadienoic | 39.99 | 0.2 |
| MOL006937 | 12,13-epoxy-9-hydroxynonadeca-7,10-dienoic acid | 42.15 | 0.24 |
| MOL006957 | (3S,6S)-3-(benzyl)-6-(4-hydroxybenzyl)piperazine-2,5-quinone | 46.89 | 0.27 |
| MOL003578 | Cycloartenol | 38.69 | 0.78 |
| MOL006967 | beta-D-Ribofuranoside, xanthine-9 | 44.72 | 0.21 |

1. : Compounds in BX

| TFL-Mol ID | Molecule Name | OB (%) | DL |
| --- | --- | --- | --- |
| MOL013117 | 4,7-Dihydroxy-5-methoxyl-6-methyl-8-formyl-flavan | 37.03 | 0.28 |
| MOL013118 | Neoastilbin | 40.54 | 0.74 |
| MOL013119 | Enhydrin | 40.56 | 0.74 |
| MOL013129 | (2R,3R)-2-(3,5-dihydroxyphenyl)-3,5, 7-trihydroxychroman-4-one | 63.17 | 0.27 |
| MOL001736 | (-)-taxifolin | 60.51 | 0.27 |
| MOL000358 | beta-sitosterol | 36.91 | 0.75 |
| MOL000359 | sitosterol | 36.91 | 0.75 |
| MOL004328 | naringenin | 59.29 | 0.21 |
| MOL000449 | Stigmasterol | 43.83 | 0.76 |
| MOL004567 | isoengelitin | 34.65 | 0.7 |
| MOL004575 | astilbin | 36.46 | 0.74 |
| MOL004576 | taxifolin | 57.84 | 0.27 |
| MOL004580 | cis-Dihydroquercetin | 66.44 | 0.27 |
| MOL000546 | diosgenin | 80.88 | 0.81 |
| MOL000098 | quercetin | 46.43 | 0.28 |

(C): Compounds in TFL
